# IGF binding protein 2 is a cell-autonomous factor supporting survival and migration of acute leukemia cells

**DOI:** 10.1186/1756-8722-6-72

**Published:** 2013-10-08

**Authors:** Xiaoli Chen, Junke Zheng, Yizhou Zou, Chun Song, Xuemei Hu, Cheng Cheng Zhang

**Affiliations:** 1 Departments of Physiology and Developmental Biology, University of Texas Southwestern Medical Center, 5323 Harry Hines Boulevard, Dallas 75390, TX, USA; 2 Department of Immunology, Central South University School of Xiangya Medicine, Changsha, China; 3 Shandong University and National New Drug R&D Center in Shandong, Jinan, China; 4 Department of Immunology, Binzhou Medical University, Yantai, China

## Abstract

**Background:**

The role of IGF binding protein 2 (IGFBP2) in cancer development is intriguing. Previously we identified IGFBP2 as an extrinsic factor that supports the activity of hematopoietic stem cells (HSCs).

**Methods and results:**

Here we investigated the role of IGFBP2 in in human leukemia cells and in the retroviral AML1-ETO9a transplantation acute myeloid leukemia (AML) mouse model.

**Results:**

IGFBP2 is highly expressed in certain human AML and acute lymphoblastic leukemia (ALL) cells. Inhibition of expression of endogenous IGFBP2 in human leukemia cells led to elevated apoptosis and decreased migration and, consistently, to decreased activation of AKT and other signaling molecules. We also studied the effects of IGFBP2 knockout in the retroviral AML1-ETO9a transplantation AML mouse model. The deletion of IGFBP2 in donor AML cells significantly decreased leukemia development in transplanted mice. Lack of IGFBP2 resulted in upregulation of PTEN expression and downregulation of AKT activation, in the mouse AML cells. The treatment of IGFBP2 deficient AML cells with a PTEN inhibitor restored the wild-type colony forming ability. The deletion of IGFBP2 also led to decreased AML infiltration into peripheral organs and tissues, suggesting that IGFBP2 is required for the migration of AML cells out of bone marrow.

**Conclusion:**

IGFBP2 is a critical cell-autonomous factor that promotes the survival and migration of acute leukemia cells.

## Introduction

Acute myeloid leukemia (AML) is characterized by rapid proliferation of immature myeloid blasts in the bone marrow. It is the most common acute leukemia affecting adults and accounts for about 1.2% of cancer deaths in the United States each year. Despite treatment, the majority of the patients relapse within 5 years [1]. To effectively treat AML, new molecular targets and therapeutic approaches need to be identified.

Insulin-like growth factor binding protein 2 (IGFBP2) is a member of the IGFBP family; this family contains at least six circulating proteins that bind IGF-1 and IGF-2 with an affinity equal or greater than that of the three IGF receptors. IGFBPs modulate the biological effects of IGFs by controlling IGF distribution, function, and activity [2,3]. IGFBP2 preferentially binds IGF-2 over IGF-1. IGFBP2 is expressed in the fetus and in a number of adult tissues and biological fluids [4].

The role of IGFBP2 in cell growth and cancer development is intriguing. While IGFBP2 can bind to IGF ligands and displays IGF-dependent growth inhibitory effects on many cell types, it also has intrinsic bioactivities that are independent of IGF-1 and IGF-2. IGFBP2 binds to the cell surface [5,6] and binds to integrin α5 [6-8] and to αv [9] extracellularly and intracellularly. It stimulates telomerase activity [10], activates MMP-2 [11], modulates MAPK activation [10], and supports proliferation, survival, differentiation, and motility of various types of cells by suppression of PTEN and activation of AKT, integrin, integrin-linked kinase (ILK), and NF-κB pathways [6-8,10,12-23]. Intracellular IGFBP2 promotes angiogenesis by stimulating VEGF transactivation [24]. In addition, oxidative stress leads to the uptake of IGFBP2 into the cell cytosol after 12–24 h [12,25].

IGFBP2 is expressed at significantly higher levels in AML patients than in healthy volunteers [26]. A lower IGFBP2 level is associated with longer-term survival of patients with AML and ALL [27,28]. Expression of IGFBP2 is also an independent factor for the prediction of relapse of AML and ALL [26,27,29,30]. Moreover, IGFBP2 is overexpressed in many patients with other tumors, and in some cases its expression correlates with grade of malignancy [6,10,12]. The level of IGFBP2 appears to be low in well-differentiated tumors but high in poorly differentiated tumors [31].

We recently identified IGFBP2 as an extrinsic factor that supports the activity of hematopoietic stem cells (HSCs) [19,32,33]. To understand the potential functional role of IGFBP2 in leukemia development, we addressed several questions in the current study: 1) Is IGFBP2 expressed by leukemia cells? If so, what is function for these cells? 2) Is IGFBP2’s effect on leukemia cells an environmental effect or cell-autonomous effect? 3) What signaling pathways are regulated by IGFBP2 in leukemia cells? We determined that IGFBP2 supports the survival and migration of acute leukemia cells in a cell-autonomous manner. IGFBP2 is essential for regulation of several signaling pathways including PTEN/AKT signaling in AML and perhaps B-ALL cells.

## Results

### *IGFBP2* is highly expressed in certain human AML cells

**Figure 1:**
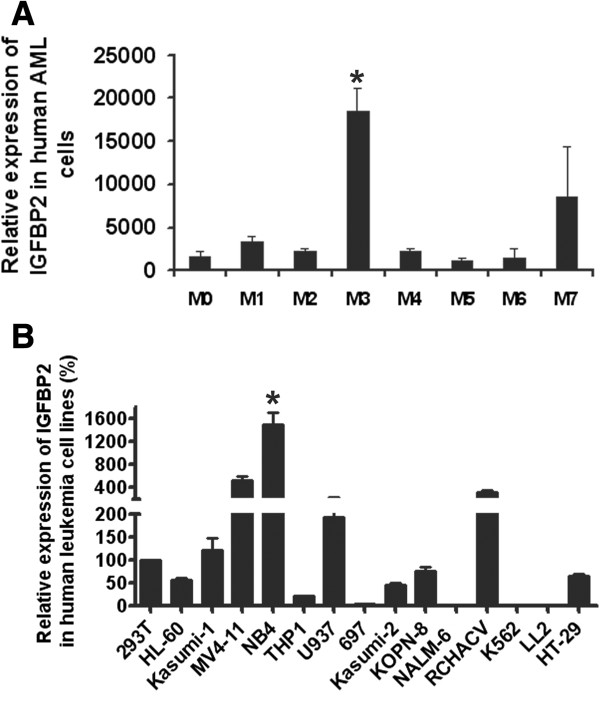
***IGFBP2 *is highly expressed in human acute myeloid leukemia cells. (A)** An *in silico* analysis of human *IGFBP2* mRNA expression in different human AML subtypes (n = 195, TCGA database; * *p* < 0.05). **(B)** Expression of *IGFBP2* in different human cancer cell lines as determined by real-time RT-PCR (n = 3). * significant different from other cell line values, *p* < 0.05.

We performed an *in silico* analysis of *IGFBP2* mRNA expression in different subtypes of human AML based on data from the TCGA AML database (http://cancergenome.nih.gov/; accessed November 5, 2012). *IGFBP2* is expressed at significantly higher levels in cells of the M3 subtype than of other subtypes tested (Figure 1A). The M3 subtype is characteristic of the acute promyelocytic leukemia (APL) [t (15;17)] that generates the fusion protein promyelocytic leukemia-retinoic acid receptor α (PML-RARA).

We further measured *IGFBP2* expression in a number of human cancer cell lines including AML and ALL lines. Although *IGFBP2* mRNA was expressed at the highest levels in the M3 subtype NB4 AML cells, it was also highly expressed in some other AML and B cell-derived ALL (B-ALL) cells including MV4-11 (M5 AML), U937 (B-ALL), and RCH-ACV (B-ALL) (Figure 1B). By contrast, it was expressed at very low levels in K562 (CML) cells and NALM-6 (B-ALL) cells (Figure 1B).

### IGFBP2 is critical for survival and migration of human AML cells

**Figure 2:**
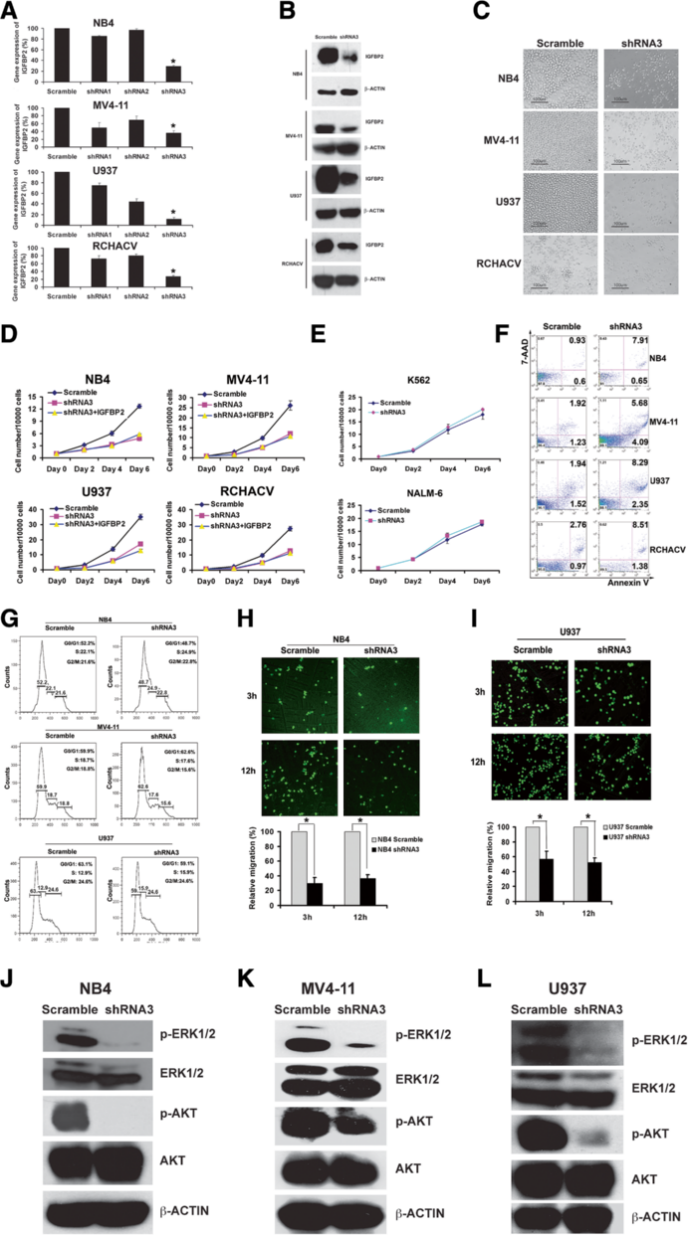
**IGFBP2 is critical for survival and migration of human AML cells. (A) ***IGFBP2* mRNA expression was reduced in four human leukemia cell lines by shRNA3 as determined by real-time RT-PCR (n = 3). * significant different from scramble control values, *p* < 0.05. **(B)** IGFBP2 levels were reduced in four human leukemia cell lines by shRNA3 as determined by western blotting (n = 3). **(C)** Inhibition of *IGFBP2* expression with shRNA3 in four human leukemia cell lines that have high IGFBP2 levels decreased cell growth in culture (n = 3). **(D)** Effects of inhibition of *IGFBP2* expression with shRNA3 cannot be rescued by extrinsic recombinant IGFBP2 protein (500 ng/ml) (n = 3). **(E)** Inhibition of *IGFBP2* expression with shRNA3 in two human leukemia cell lines that have low IGFBP2 levels did not decrease cell growth in culture (n = 3). **(F)** Inhibition of *IGFBP2* expression with shRNA3 in four human leukemia cell lines that have high IGFBP2 levels induced apoptosis as determined by Annexin v and 7-AAD staining in flow cytometry. **(G)** Inhibition of *IGFBP2* expression with shRNA3 did not induce cell cycle arrest in AML cell lines as determined by propidium iodide staining and analysis by flow cytometry. **(H-I)** Inhibition of *IGFBP2* expression with shRNA3 suppressed NB4 and U937 cell migration. Indicated cells transduced with scrambled shRNA or shRNA3 were placed in the upper chamber of a transwell insert (5-μm pore size). Cells were allowed to migrate for 3 or 12 hours at 37°C before harvesting and analysis (n = 3; ** p < 0.01). The migration was normalized by the cell numbers at 12 hr. **(J-L)** Inhibition of *IGFBP2* expression with shRNA3 decreased the activation of ERK and AKT in NB4, MV4-11, and U937 cell lines.

We studied the potential function of IGFBP2 in human leukemia cells by silencing its expression with lentivirus encoded small hairpin RNAs (shRNAs). Three previously reported RNAi sequences targeting *IGFBP2* mRNA [34] were evaluated; shRNA3 efficiently decreased expression of *IGFBP2* mRNA (Figure 2A) and IGFBP2 protein (Figure 2B), concordant with a previous report [35]. Importantly, the inhibition of *IGFBP2* expression effectively inhibited the in vitro growth of NB4, MV4-11, U937, and RCHACV cells that express high endogenous *IGFBP2* levels (Figure 2C-D) but had little effect on K562 cells or NAML-6 cells that have extremely low levels of *IGFBP2* expression (Figure 2E). The inclusion of extrinsic recombinant IGFBP2 in the culture medium did not rescue the defects in leukemia cells treated with shRNA targeting *IGFBP2* (Figure 2D). This result suggests that leukemia cells behave differently from HSCs and certain solid cancer cells. While IGFBP2 has cell-autonomous effect to support growth of leukemia cells, the extrinsic IGFBP2 stimulates the activity of HSCs and some other cancer cells such as breast cancer cells [19,23,33].

To determine the underlying mechanism by which IGFBP2 supports the growth of leukemia cells, we compared levels of apoptosis and cell cycle status of AML cells treated with shRNA3 or scrambled control shRNA. Cells treated with the shRNA targeting *IGFBP2* had increased levels of early and late apoptosis compared to cells treated with the control shRNA (Figure 2F; 0.60%, 1.23%, 1.52%, and 0.97% early apoptotic cells in controls vs. 0.65%, 4.09%, 2.35%, and 1.38% in knockdown cells, and 0.93%, 1.92%, 1.94%, and 2.76% late apoptotic cells in controls vs. 7.91%, 5.68%, 8.29%, and 8.51% in knockdown cells, at day 6 of culture). In contrast, there was no significant difference in cell cycle distribution between cells treated with control shRNA and shRNA targeting *IGFBP2* (Figure 2G). Furthermore, we observed that inhibition of *IGFBP2* expression in NB4 or U937 cells decreased cell migration in a transwell assay (Figure 2H-I).

We next examined whether ERK and AKT signaling are involved in the effects of IGFBP2 on these leukemia cells. Compared to control treated cells, NB4, MV4-11, and U937 cells treated with shRNA targeting *IGFBP2* had significantly decreased phosphorylation of ERK and AKT (Figure 2J-L). We also observed increased levels of PTEN in NB4 cells (Additional file 1: Figure S1). These results suggest that, as observed in other systems [6-8,10,12-22], ERK and PTEN/AKT signaling pathways are possible effectors of IGFBP2 in human leukemia cells. Together, our results suggest that IGFBP2 has cell-autonomous effects on leukemia cells and is critical for their survival and migration.

### IGFBP2 supports leukemia development in the mouse AML model

To gain a deeper understanding of the mechanism by which IGFBP2 supports AML development, we studied AML development in *IGFBP2*-null mice. While IGFBP2 is expressed at high levels by M3 t(15:17) APL cells that produce a fusion protein promyelocytic leukemia-retinoic acid receptor α (PML-RARA), the physiologic PML-RARA expression from the mouse *pml* locus rarely causes leukemia development [36]. IGFBP2 is also highly expressed in AML1-ETO cells (Additional file 1: Figure S2) [37], which do cause leukemia development in a transplant model [38]. We, therefore, sought to use *IGFBP2*-null mice to study how IGFBP2 affects AML development in the AML1-ETO9a (AE9a) retroviral transplantation mouse model [38].

**Figure 3:**
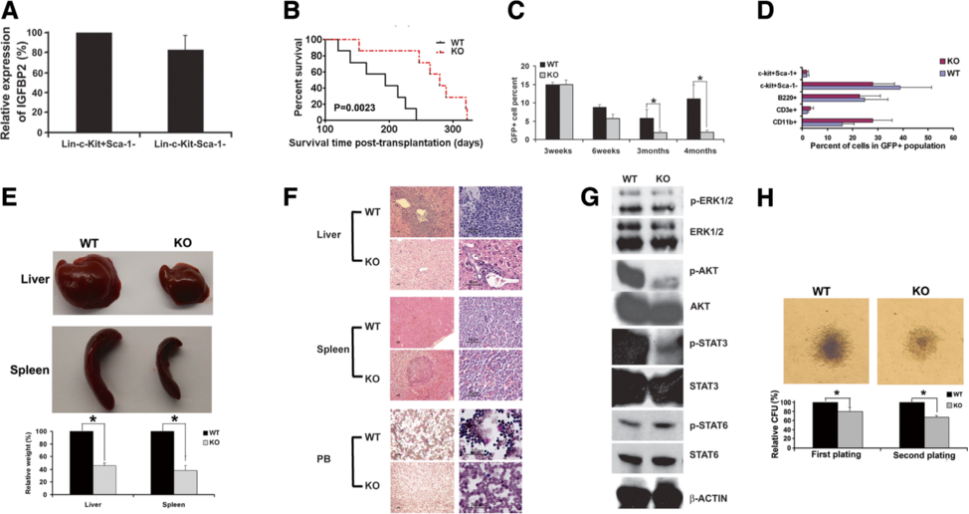
**Inhibition of *IGFBP2 *expression delays leukemia development in the AML1-ETO transplantation mouse AML model. (A)** Expression of IGFBP2 in AML1-ETO infected BM Lin^-^Kit^+^Sca-1^-^ cells and Lin^-^Kit^-^Sca-1^-^ cells as determined by real-time RT-PCR (n = 3). **(B)** Mice transplanted with AML1-ETO-infected wild-type hematopoietic progenitors had significantly reduced survival compared to that of mice transplanted with AML1-ETO-infected *IGFBP2*-null hematopoietic progenitors (n = 14; *p* < 0.005). **(C) ***IGFBP2*-null AML mice had fewer leukemic GFP^+^ cells in peripheral blood than mice transplanted with wild-type cells at 3–4 months after transplantation (n = 14; * *p* < 0.05). **(D)** Percentages of progenitors and differentiated cells in the control and *IGFBP2*-null leukemic GFP^+^ compartments of peripheral blood at four months after transplantation (n = 14). **(E)** Comparison of the sizes of spleens and livers of the mice transplanted with wild-type AML1-ETO9a cells and those transplanted with *IGFBP2*-null AML1-ETO9a cells at 4 month after transplantation (* *p* < 0.05). **(F)** Histological hematoxylin/eosin staining of AML infiltration in the livers and spleens and Wright-Giemsa staining of peripheral blood cells of mice transplanted with control or *IGFBP2*-null AML1-ETO9a cells. **(G)** Phosphorylation of AKT and STAT3 decreased in *IGFBP2*-null AML BM cells compared with the levels in control cells. **(H)** The *IGFBP2*-null AML BM cells from primary transplant mice showed significantly decreased colony forming ability (n = 3) and morphology changes relative to cells from mice transplanted with wild-type AML cells (n = 3, * *p* < 0.05).

In drastic contrast to the observation that normal HSCs do not show a detectable *IGFBP2* mRNA level and differentiated hematopoietic cells express significant amount of IGFBP2 [19], *IGFBP2* is highly expressed in both AML stem cells (AML-SC) enriched bone marrow Lin^-^Kit^+^Sca-1^-^ cells [38] and differentiated Lin^-^Kit^-^Sca-1^-^ cells in the AML1-ETO9a AML model (Figure 3A). The mice transplanted with the AE9a-transduced *IGFBP2*-null cells developed AML significantly more slowly than controls transplanted with wild-type cells (Figure 3B). All mice transplanted with control AE9a AML cells died within 240 days post-transplantation. In contrast, more than 80% of mice transplanted with *IGFBP2*-null counterparts survived longer than 240 days (Figure 3B). The percentages of wild-type GFP^+^ AML cells were significantly greater than the null counterparts after 3 months post-transplantation (Figure 3C). However, within the GFP^+^ leukemia compartments, there were no significant differences in the AML-SC population or in the more differentiated Mac-1^+^, B220^+^, or CD3^+^ cells in mice that received *IGFBP2*-null cells and in those transplanted with wild-type cells based on flow cytometry analyses (Figure 3D). Mice transplanted with *IGFBP2*-null AML cells had significantly decreased liver and spleen sizes than mice transplanted with wild-type cells at 4 months post-transplantation (Figure 3E). The examination of leukemia infiltration into spleen and liver also revealed that *IGFBP2*-null AML cells less effectively induced leukemia than wild-type AML cells (Figure 3F). The analysis of key signaling molecules revealed that deficiency of IGFBP2 decreased the levels of phosphorylated forms of AKT and STAT3 whereas those of ERK and STAT6 remained unaffected (Figure 3G). Colony forming unit (CFU) assays showed that knockout of *IGFBP2* led to a 20% decrease in CFUs in the primary plating, and more than 30% of decrease in CFUs in the secondary plating (Figure 3H), indicating that IGFBP2 enhances self-renewal of AML cells in vitro. Deficiency of IGFBP2 also altered the morphology of colonies (Figure 3H, upper panels).

**Figure 4:**
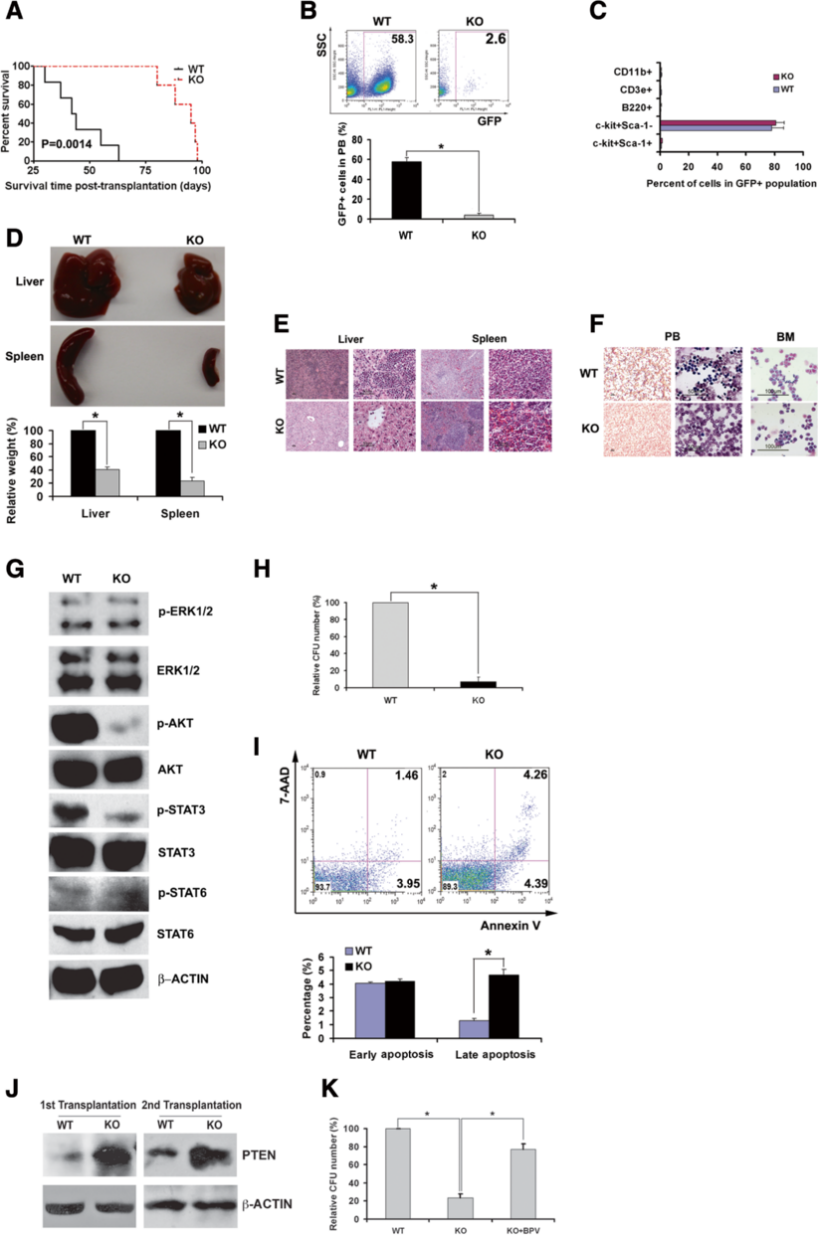
**Knockout of *IGFBP2 *delays leukemia development in the AML1-ETO AML model upon serial transplantation. (A)** Mice transplanted with AML1-ETO-infected wild-type hematopoietic progenitors had significantly reduced survival upon secondary transplantation compared to that of mice transplanted with *IGFBP2*-null cells (n = 11; *p* < 0.01). **(B) ***IGFBP2*-null AML mice had significantly fewer leukemic GFP^+^ cells in peripheral blood compared to mice transplanted with wild-type cells at 3 weeks after secondary transplantation (n = 11; * *p* <0.05). **(C)** Percentages of progenitors and differentiated cells in the control and *IGFBP2-*null leukemic GFP^+^ compartments of peripheral blood at 3 weeks after secondary transplantation (n = 10). **(D)** Comparison of the sizes of spleens and livers of the mice transplanted with wild-type and *IGFBP2*-null AML1-ETO9a cells at 1 month after secondary transplantation (* *p* < 0.05). **(E)** Histological hematoxylin/eosin staining of AML infiltration in the livers and spleens of mice secondarily transplanted with control or *IGFBP2*-null AML1-ETO9a cells. **(F)** Representative Wright-Giemsa staining of peripheral blood and bone marrow AML cells from leukemic mice after secondary transplantation. **(G)** Phosphorylation of AKT and STAT3 decreased in *IGFBP2*-null AML BM cells compared with levels in the control cells. **(H) ***IGFBP2*-null AML1-ETO9a BM cells from secondarily transplanted mice had dramatically decreased CFU forming ability, including decreased colony number and size, relative to wild-type AML1-ETO9a BM cells (n = 6; * *p* < 0.05). **(I) ***IGFBP2*-null AML1-ETO9a BM cells showed increased apoptosis relative to control cells (n = 6; * *p* < 0.05). **(J)** AML cells from *IGFBP2*-null BM showed higher levels of PTEN expression than those from control mice both in primary and secondary transplantation. **(K)** A PTEN inhibitor increased the CFU activity of *IGFBP2*-null AML cells (n = 3; * *p* < 0.05).

We performed secondary transplantation to investigate the role of IGFBP2 in the activity of mouse AML-SCs. The *IGFBP2-*null bone marrow AML cells decreased AML development during secondary transplantation (Figure 4A-F) and had dramatically decreased AKT and STAT3 activation (Figure 4G) as was observed in the primary transplanted cells. *IGFBP2-*null cells resulted in only 5% of the CFU of wild-type AML cells (Figure 4H). Importantly, similar to the human leukemia cells, the deletion of *IGFBP2* induced increased apoptosis of mouse bone marrow AML cells (Figure 4I). In both primary and secondary transplantation, PTEN levels were increased in *IGFBP2*-null AML cells relative to levels in wild-type cells (Figure 4J). Importantly, the PTEN inhibitor bpV(HOpic) was capable of rescuing the CFU defects of null bone marrow AML cells (Figure 4K). Therefore, IGFBP2 supports the survival of AML-SCs, and PTEN/AKT signaling and STAT3 signaling may play roles in IGFBP2-regulated AML-SC activity.

### IGFBP2 supports the mobilization of mouse AML cells

**Figure 5:**
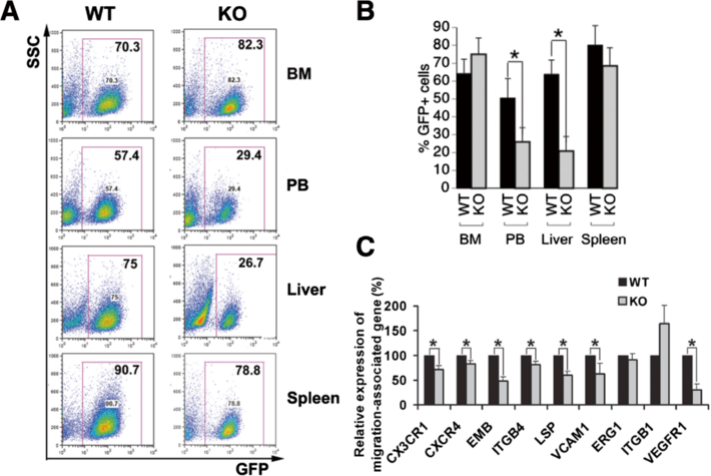
**Knockout of *IGFBP2 *decreases migration of leukemia cells out of bone marrow in the AML1-ETO mouse AML model. (A-B)** Representative flow cytometry plots **(A)** and summary of results **(B)** showing the mice transplanted with *IGFBP2*-null AML cells have significantly decreased GFP^+^ AML cells in peripheral blood, liver, and spleen but not in BM compared with the mice transplanted with control AML cells. **(C)** The *IGFBP2*-null AML BM cells have decreased expression of a number of surface proteins compared to wild-type cells (n = 3; * *p* < 0.05).

Because IGFBP2 plays a role in cell migration [7,11,12,20], we compared the distribution of wild-type and *IGFBP2-*null AML cells in bone marrow and peripheral tissues and organs. Although we found that the percentages of *IGFBP2-*null AML cells did not differ from those of wild-type cells in BM, the existence of *IGFBP2-*null AML cells in peripheral blood, spleen, and liver was significantly decreased relative to levels of wild-type AML cells (Figure 5A-B). Concordantly, the expression of surface proteins (CX3CR1, CXCR4, EMB, ITGB4, LSP, VCAM1) important for leukemia infiltration was downregulated in *IGFBP2-*null bone marrow AML cells (Figure 5C). Together with the migration enhancing effect of IGFBP2 in human leukemia cells (Figure 2H-I), our results suggest that IGFBP2 enhances the mobilization of AML cells, thus accelerating AML development.

## Discussion

Previously, we showed that IGFBP2 stimulates the activities of mouse and human HSCs in vitro and in vivo [19,32,39]. Here, we demonstrated that, 1) IGFBP2 is highly expressed by certain types of acute leukemia cells, 2) IGFBP2 is a cell-autonomous factor that promotes the development of acute leukemia, 3) IGFBP2 supports both survival and migration of leukemia cells, and 4) the stimulating effect of IGFBP2 on acute leukemia cells depends on PTEN signaling. To our knowledge, this is the first functional demonstration that IGFBP2 is critical for leukemia development. Our results are concordant with reports that IGFBP2 is considered a prognostic factor for AML and ALL [26,27,29,30] and activates AKT [7] and suppresses PTEN expression [22,23] in certain solid cancer cells.

It is rather surprising that IGFBP2, a non-essential factor for normal development, is detrimental for acute leukemia cells upon deletion. Our study suggests that the different effects of IGFBP2 on normal HSCs and leukemia cells contribute to this phenomenon. Normal HSCs express little IGFBP2 per se [19], whereas both leukemia stem cells and differentiated leukemia cells have similar high expression of IGFBP2. Consistently, the extrinsic IGFBP2 has a supporting effect on HSC expansion [19,33], and intrinsic IGFBP2 promotes the survival and migration of AML cells (including both AML–SCs and differentiated AML cells) in a cell-autonomous manner. Inhibition of *IGFBP2* expression in human leukemia cell lines effectively inhibited growth of these cells. Importantly, the exogenous recombinant IGFBP2 added to the culture medium did not have a potent rescue effect. In addition, HSCs and acute leukemia cells represent different cell identities and likely have very different signaling networks and thus use distinctive mechanisms to utilize IGFBP2. While a major question in IGFBP biology is whether the effect of IGFBP2 is environmental or cell-autonomous, our studies on HSCs and leukemia indicated that the answers are cell-type-dependent.

The cell-autonomous effect of IGFBP2 in leukemia cells is also different from the extrinsic effect of IGFBP2 in supporting survival of certain solid cancer cells such as breast cancer cells [23]. Nevertheless, it has been shown that intrinsic IGFBP2 interacts with integrin α5β1 and promotes migration of glioma cells and glioma progression through activation of AKT, ILK, and NF-κB pathways [7,8,20]. Moreover, endogenous IGFBP2 stimulates the transactivation of VEGF and supports angiogenesis [24]. Concordantly, here our study suggested that the AKT pathway in leukemia cells plays a critical role in the effects of IGFBP2, and we demonstrated that the PTEN inhibitor treatment rescues the colony forming activity of the IGFBP2 deficient leukemia cells. Overall, the diverse IGFBP2 actions possibly result from the different cell identities that have distinctive extracellular or intracellular IGFBP2-interacting molecules, and the IGFBP2 related signaling in different cells may be quite different. Indeed, consistent with the different expression of IGFBP2 in HSCs and leukemia cells, the signaling defects we observed in IGFBP2 deficient leukemia cells are more dramatic than in IGFBP2-null HSCs, and also are unique compared to defects observed in other cancer cells upon IGFBP2 deletion [23]. Further investigations are warranted to determine how IGFBP2 has different effects on normal cells and various types of cancer cells.

## Conclusion

Here we showed that IGFBP2 was required for both of survival and migration of AML and ALL cells. The inhibition of *IGFBP2* expression in human AML and B-ALL cell lines increased apoptosis and decreased migration, and these results were confirmed in vivo using the *IGFBP2*-null AML1-ETO9a model. These novel data indicated that IGFBP2 supports leukemia development autonomously by both enhancing cell survival and promoting migration out of bone marrow and infiltration into peripheral organs and tissues. The ability of IGFBP2 to support cancer cell survival or migration has been documented in other cancer cell types. For example, IGFBP2 has anti-apoptotic effects in multiple types of solid cancer [7,12,13,23], binds to integrin α5 resulting in migration of Ewing’s sarcoma cells [6], and activates integrin β1 to induce glioma cell motility [20]. Because IGFBP2 is not expressed by normal HSCs but highly expressed by leukemia stem cells and differentiated leukemia cells, it is desirable to develop anti-IGFBP2 therapy that may effectively induce apoptosis and block mobilization of leukemia cells including leukemia stem cells with minimal toxicity to normal HSCs.

## Methods

### Mice, shRNAs, and primers

C57BL/6 CD45.2 and CD45.1 mice were purchased from the National Cancer Institute and the University of Texas Southwestern Medical Center animal breeding core facility. The IGFBP2^+/−^ mice in C57BL/6 background were previously described [19]. Mice were maintained at the University of Texas Southwestern Medical Center animal facility. All animal experiments were performed with the approval of UT Southwestern Committee on Animal Care. Western blots were performed to detect the IGFBP2 protein using a goat anti-IGFBP2 antibody (SC-6002; Santa Cruz Biotechnology). The sequences for the shRNAs and RT-PCR primers for human IGFBP2 are listed below.

Scramble shRNA: 5′-GATATGTGCGTACCTAGCAT-3′

IGFBP2 shRNA1: 5′-AATGGCGATGACCACTCAGAA-3′

IGFBP2 shRNA2: 5′-GATATGTGCGTACCTAGCAT-3′

IGFBP2 shRNA3: 5′-ACTGTGACAAGCATGGCCTGT-3′

Human IGFBP2 Forward Primer: 5′-GCCCTCTGGAGCACCTCTACT-3′

Human IGFBP2 Reverse Primer: 5′-CATCTTGCACTGTTTGAGGTTGTAC-3′

### Retroviral infection and transplantation

Human embryonic kidney 293 T cells were grown in DMEM with 10% fetal bovine serum (FBS) and transfected with an MSCV-AML1-ETO9a-IRES-GFP encoding plasmid [38] and pCL-ECO to produce retroviruses. The infection of Lin^-^ cells with retrovirus was performed as described previously [40]. Briefly, we incubated Lin^-^ cells overnight in medium with 10% FBS, 20 ng/mL SCF, 20 ng/ml IL-3, and 10 ng/mL IL-6, followed by spin infection with retroviral supernatant in the presence of 4 μg/mL polybrene. Infected cells (300,000) were transplanted into lethally irradiated (1000 rad) C57BL/6 mice by retro-orbital injection. For secondary transplantation, GFP^+^ bone marrow (BM) cells from primary transplanted mice were transplanted into mice with 100,000 normal BM cells as competitors.

### Flow cytometry, immunohistochemistry, and cytospin

Flow cytometry, immunohistochemistry, and cytospin were performed as we described previously [40]. For flow cytometry analysis of AML cells, peripheral blood or BM cells were stained with anti-Lineage-Biotin (followed by streptavidin-APC), anti-Mac-1-APC, anti-Gr-1-PE, anti-CD3-APC, anti-B220-PE, or anti-Kit-PE monoclonal antibodies (BD Pharmingen). Cell cycle status was determined by propidium iodide staining. For analysis of apoptosis, cells were stained with PE-conjugated anti-annexin V and 7-AAD (BD Pharmingen) according to the manufacturer’s instructions.

### Colony forming unit (CFU) assays

Cells from AML mice were plated in methylcellulose (M3534, Stem Cell Technologies) for CFU-GM assays, according to the manufacturer’s protocols and our previously published protocol [40,41]. After 7 days, 2000 cells from three dishes were used for secondary replating. 1 μM PTEN inhibitor bpV(HOpic) (CalBiochem) was used to treat AML cells for the CFU assay as indicated.

### Western blotting

Cell lysates (100 μg samples) were separated by electrophoresis on a 4-12% SDS-polyacrylamide gel, and the proteins were electroblotted onto a nitrocellulose membrane. The membrane was probed with primary antibody for 1 h at room temperature and then incubated with horseradish peroxidase-conjugated secondary antibody, which was detected with the chemiluminescence SuperSignal kit (Pierce).

### Quantitative RT-PCR

Total RNA was isolated from FACS-collected cells. First-strand cDNA was synthesized using SuperScript II RT (Invitrogen). Samples were analyzed in triplicate 25-μl reactions (300 nM each primer, 12.5 μl of Master mix) as adapted from the standard protocol provided in SYBR Green PCR Master Mix and RT-PCR Protocols provided by Applied Biosystems. Primers were purchased from Qiagen or Sigma. The default PCR protocol was used on an Applied Biosystems Prism 7000 Sequence Detection System. The mRNA level in each population was normalized to the level of *β-actin* RNA transcripts present in the same sample as described previously [39].

### Statistical analyses

Data are expressed as mean ± SEM. Data were analyzed by Student’s *t* test and were considered statistically significant if *p* < 0.05. The survival rates of the two groups were analyzed using a log-rank test.

## Abbreviations

ALL: Acute lymphoblastic leukemia; AML: Acute myeloid leukemia; HSCs: Hematopoietic stem cells; IGF: Insulin-like growth factor; IGFBP2: IGF binding protein 2; ILK: Integrin-linked kinase; PTEN: Phosphatase and tensin homolog; WT: Wild-type.

## Competing interest

The authors declare no competing financial interests.

## Authors’ contributions

XC, JZ, and CCZ contributed to design, performed experiments, interpreted data, and contributed to writing of the manuscript. YZ, CS, and XH contributed to experimental performance and interpretation. All authors read and approved the final manuscript.

## Authors’ information

C.C.Z. is an associate professor at UT Southwestern Medical Center, focusing on the roles of secreted proteins and cell surface receptors in the ex vivo expansion of hematopoietic stem cells and leukemia development.

## Supplementary Material

Additional file 1: Figure S1 PTEN levels were increased in NB4 cells treated with shRNA targeting *IGFBP2*. **Figure S2.** An *in silico* analysis of *IGFBP2* expression in AML1-ETO transduced CD34+ human cord blood or peripheral blood cells based on the published database in reference 37. Click here for file
