# Supplementary material for: IGF binding protein 2 is a cell-autonomous factor supporting survival and migration of acute leukemia cells
Source: J Hematol Oncol. 2013 Oct 8;6:72. doi: 10.1186/1756-8722-6-72 (PMC3851819; doi:10.1186/1756-8722-6-72)
Supplement: Additional file 1: Figure S1 — PTEN levels were increased in NB4 cells treated with shRNA targeting IGFBP2. Figure S2. An in silico analysis of IGFBP2 expression in AML1-ETO transduced CD34+ human cord blood or peripheral blood cells based on the published database in reference 37. [file 1756-8722-6-72-S1.pdf]

Supplementary Materials

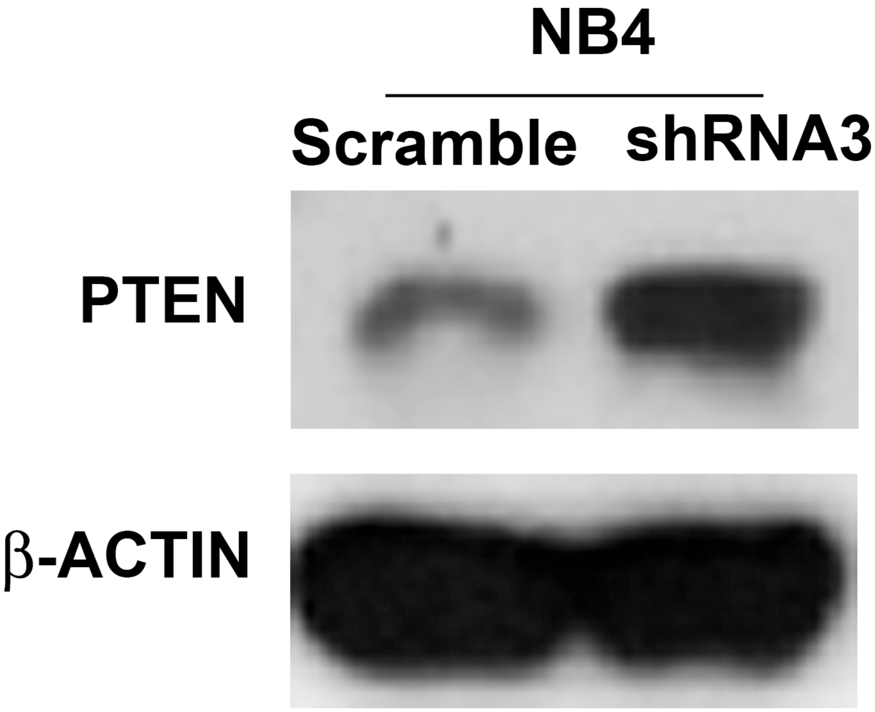

sFigure 1. PTEN levels were increased in NB4 cells treated with shRNA targeting *IGFBP2*.

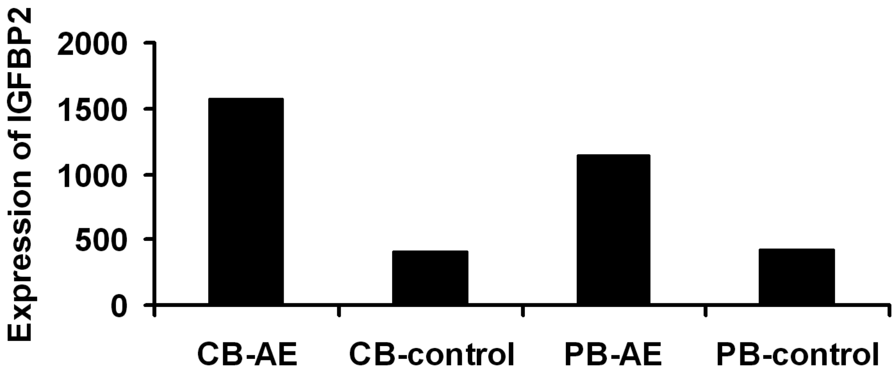

sFigure 2. An *in silico* analysis of *IGFBP2* expression in AML1-ETO transduced CD34$^+$ human cord blood or peripheral blood cells based on the published database [40].
